# A Clinical Paradigm for Listening Effort Assessment in Middle-Aged Listeners

**DOI:** 10.3389/fpsyg.2022.820227

**Published:** 2022-02-17

**Authors:** Ricky Kaplan Neeman, Ilan Roziner, Chava Muchnik

**Affiliations:** ^1^Department of Communication Disorders, The Stanley Steyer School of Health Professions, Sackler Faculty of Medicine, Tel Aviv University, Tel Aviv, Israel; ^2^Hearing, Speech and Language Center, Sheba Medical Cente, Ramat-Gan, Israel

**Keywords:** listening effort, dual-task paradigm, middle- aged adults, cognitive cost, a clinical paradigm

## Abstract

Listening effort (LE) has been known to characterize speech recognition in noise regardless of hearing sensitivity and age. Whereas the behavioral measure of dual-task paradigm effectively manifests the cognitive cost that listeners exert when processing speech in background noise, there is no consensus as to a clinical procedure that might best express LE. In order to assess the cognitive load underlying speech recognition in noise and promote counselling for coping strategies, a feasible clinical paradigm is warranted. The ecological validity of such a paradigm might best be demonstrated in middle-aged adults, exhibiting intact hearing sensitivity on one hand, however, experiencing difficulties in degraded listening conditions, unaware of the implicated cognitive cost of speech recognition in noise. To this end, we constructed a dual-task paradigm that consists of a primary task of sentences-in-noise recognition and a secondary task of simple visual colored-shape matching. Research objective was to develop a clinical paradigm for the assessment of LE in middle-aged adults. Participants were 17 middle-aged adults (mean age of 52.81 years) and 23 young adults (mean age of 24.90 years). All participants had normal hearing according to age. Speech stimuli consisted of the Hebrew Matrix sentences in noise test. SRTn was obtained for 80% correct identification. Visual stimuli were colored geometric shapes. Outcome measures were obtained initially for each task separately, to establish performance ability, and then obtained simultaneously. Reaction time and accuracy in the secondary task were the defined metrics for LE. Results: LE was indicated for both groups, however, was more pronounced in the middle-aged, manifested in the visual accuracy and reaction time metrics. Both groups maintained the 80% correct recognition-in-noise in the dual-task, however, the middle-aged group necessitated a better SNR of 1.4dB than the normal hearing group. Moreover, the middle-aged group was taxed in a greater prolongation of reaction time, in order to uphold the correct recognition. Conclusion: a dual-task paradigm consisting of sentences-in-noise primary task combined with a simple secondary task successfully showed different manifestations of LE in middle-aged adults compared to young adults, thus approximating the use of such a paradigm in a clinical setting.

## Introduction

Unraveling the difficulty of speech recognition in background noise has been a major challenge in hearing research for many years. The cause-effect relationship is still under investigation. In addition to speech stimuli attributes, masker noise types, and various characteristics of the listener, such as hearing sensitivity and age (e.g., Dubno, 2015), the cognitive component has been established as a key factor in the challenge (e.g., Gordon-Salant and Samuels Cole, 2016). The ability to suppress irrelevant, distracting context and focus on desired target information is essential for speech understanding in noise (Pichora-Fuller et al., 2016). Moreover, the listener sometimes is required to perform several tasks concurrently, while ignoring background noise (Gagné et al., 2017), and, therefore, is faced with a greater cognitive load (Peele, 2018). The cognitive cost that the listener is burdened with in such complex situations is termed: “listening effort” (LE). As stated by Pichora-Fuller et al. (2016), listening effort refers to “the deliberate allocation of mental resources to overcome obstacles in goal pursuit when carrying out a listening task”. Demanding listening conditions on one hand, and increased motivation to overcome the distractions on the other hand, will affect the extent of cognitive resources allocated toward accomplishment of the target task.

Accumulating evidence shows increase in LE in the elderly (Tun et al., 2009; Gosselin and Gagné, 2011; Sommers and Phelps, 2016). As both cognitive ability and peripheral auditory function are known to decline with age (Tremblay and Backer, 2016), it is expected that older adults will exert more LE than young adults. In the middle-age (MA), on the other hand, it is more difficult to pre-establish expectations. Hearing acuity, as well as other auditory processing abilities, might not decline at the same manner. Whereas MA adults might not exhibit pure-tone thresholds elevation in the audiogram (Helfer et al., 2017), they were found to have complaints concerning their ability to understand speech in noise (Lee et al., 2015; Helfer et al., 2017). This finding was supported by research studies’ evidence of deteriorated speech perception in noise (e.g., Lee et al., 2015; Goossens et al., 2017). In an attempt to explain these MA-related speech perception difficulties in the presence of normal hearing thresholds, it was assumed that temporal processing deficiencies might underlie some of these difficulties. Indeed, behavioral studies have found reduced supra-threshold temporal auditory processing capacities (Helfer and Vargo, 2009; Füllgrabe, 2013). Moreover, electrophysiological data demonstrated neural encoding deficits of temporal fine structure in participants aged 51-67 years (Clinard and Cotter, 2015). Nonetheless, the contribution of cognitive factors was argued to serve as a fundamental aspect in the decline of speech recognition in noise in the middle-aged (Helfer et al., 2017). Studies concerning LE in MA adults might shed more light on cognitive demands of speech recognition in noise. These studies, however, are scarce. Typically, LE in the MA group was studied as a part of a large age range of normal-hearing participants (e.g., Degeest et al., 2015), or in hearing-impaired participants (e.g., Desjardins and Doherty, 2013). Degeest et al. (2015) were among the first and few researchers that explored the effect of age on LE, in a group of 60 adults, aged 20-77 years. The primary task was recognition of digits-in-noise, and the secondary task required visual memory of the position of geometric figures on a screen. In order to rule out hearing sensitivity, the authors equated the experiment listening conditions, controlling for effects of differential speech intelligibility scores. Results showed that LE increased initially in the fourth decade of life and was related to the cognitive attribute of speech recognition in degraded listening conditions. Devesse et al. (2020) were among the few studies that focused specifically on participants in the age range of 45-60 years. The performance of 29 middle-aged adults was compared to that of 35 young adults in auditory-visual speech-in noise task that combined dual, triple, and quadruple secondary tasks, to approximate real life situations. Middle-aged adults were found to perform worse than the young adults in all tasks. Their findings highlighted the difficulties of speech in noise understanding of MA adults and their need to allocate cognitive resources in order to meet speech understanding in noise requirements.

Owing to the fact that speech recognition in noise partakes a fundamental role in audiological assessment, alongside with established data concerning age-dependent difficulties in speech in noise recognition, the need for integrating LE measures in the clinic emerges. A clinical measure of LE might demonstrate the listener’s taxed cognitive capacity and provide means to identify the need for specific counseling and rehabilitation procedures (McGarrigle et al., 2014). Furthermore, such a measure might elucidate aspects of hearing disability, not yet manifested in hearing thresholds and correct recognition of speech stimuli (Lewis et al., 2016; Gagné et al., 2017; Alhanbali et al., 2019). A clinical measure of LE could be used when traditional speech perception tests result in ceiling effect (Houben et al., 2013), and might support hearing aids fitting by adequate adaptation of specific features that reduce LE (Hornsby, 2013) as well as help select a best-fit cochlear implant program (Pals et al., 2013).

Measures of LE vary among studies. Pupillometry was suggested as a sensitive measure reflecting the cognitive load encountered by the adult listener (Peele, 2018), however, dual-task measures might prove logistically more feasible for the clinical setting. In addition, as performing another task while processing speech is a ubiquitous situation, dual-task paradigms hold ecological validity (Gagné et al., 2017). Despite the great variability of dual-task experimental procedures described in the literature, there is no consensus as to a clinical procedure that might best express LE. The idea that LE is manifested in the secondary task measures led several researchers to characterize the appropriate secondary task that might best demonstrate the cognitive load inflicted upon the listener, in certain speech recognition in noise conditions. It has been suggested that a simple secondary task might not elicit the use of cognitive resources, but rather induce adaptation and habituation (Hasher and Zacks, 1979). For example, it has been shown that very little, or no change at all, was evident in LE while using a simple secondary task that required a button-press response when a red rectangle appeared on a screen. Conversely, a secondary task that demanded semantic judgment of noun recognition yielded increased sensitivity to LE (Picou et al., 2013; Picou and Ricketts, 2014). Alternatively, Ward et al. (2017) found that a visual monitoring task involving a key-press when a gray-scale image occurred twice (in a sequence of 206 images), demanded the use of cognitive processes, and LE was exhibited in the dual-task condition. Therefore, while task complexity might not solely indicate its compatibility for a secondary task, task modality also might influence dual-task performance and in turn, the allocation of cognitive resources appropriately. As denoted by Kahneman (1973), when both tasks, the primary and the secondary, draw resources from the same resource pool, performance in the primary task might be compromised. Kim et al. (2005) demonstrated increased interference in a Stroop meaning-comparison primary task, when the secondary task demanded recall of Korean verbal characters (letters). Accordingly, when both tasks engaged the phonological loop (Baddeley et al., 1998), the same limited resource pool interfered in the primary task performance. By contrast, a secondary task from a different modality, might prompt reallocation of unused resources with available reserve capacity. This idea is substantiated by studies using various visual secondary tasks that did not affect primary speech recognition in noise tasks (e.g., Hughes and Galvin, 2013; Ward et al., 2017), consistent with domain-specific attentional resources assumptions (e.g., Baddeley and Logie, 1999). Accordingly, primary and secondary tasks pertaining to different domains might better manifest LE, while preserving primary task performance (Grieco-Calub et al., 2017).

In face of the very few studies that investigated LE in the middle age specifically, and the need to incorporate LE in the audiology clinic, the purpose of the current study was to develop a clinical paradigm for the assessment of LE in middle-aged normal hearing (age-dependent) adults. In order for the paradigm to be well-suited to the clinical setting, and at the same time approximate real-life situations, the primary task consisted of sentences recognition-in-noise, and the secondary task was a simple, visual, basic shape-matching task.

## Materials and Methods

### Participants

Twenty-three young female adults (range 21.33-28.34 years, mean = 24.90, *SD* = 1.86) and 17 middle-aged (seven males, ten females) adults (range 42.33-65.90 years, mean = 52.81, *SD* = 7.76) participated in the study. All participants self-reported no history of ear diseases, used Hebrew as their primary language, did not present attention disorders, and had no experience in hearing-in-noise experiments. Hearing thresholds in the young group did not exceed 15dBHL at octave frequencies from 0.25 through 8 kHz. In the middle-aged group, hearing thresholds were normal to age (in accordance with the 75th percentile: Engdahl et al., 2005) at the same frequencies. All participants were volunteers, and signed an informed consent form prior to data collection. The study was approved by the Institutional Review Board at Tel Aviv University.

### Stimuli

**Speech stimuli** consisted of the Hebrew version of the Matrix sentences in noise test (Bugannim et al., 2019). Speech reception threshold in noise (SRTn) was obtained for the 80% of the words that were repeated correctly, using an adaptive procedure. Background noise was steady-state, test-specific, speech shaped noise, generated by superimposition of all sentences, presented at a fixed level of 60dBSPL. Sentences and noise were presented at initial SNR of 0dB, followed by increase or decrease of sentences level, depending on listeners correct word recognition.

**Visual stimuli** were three geometric shapes: squares, triangles and circles, in the colors of red, green and yellow (Hughes and Galvin, 2013). A colored shape was presented on a touch-screen for 0.5 second, followed by four colored shapes: the test shape and three foils. Participants had to touch the test shape they saw earlier.

### Testing Apparatus

Testing was conducted in a sound-attenuating room. Participants sat on a chair, facing a loudspeaker located at a distance of one meter, 0° azimuth. Speech stimuli were presented from a Toshiba Satellite Pro laptop, routed through Auritec GmbH Earbox 3.0 sound card. Visual stimuli were displayed on a Sony S1 9.4” touchscreen tablet held by the participants, who indicated their response by touching the selected matched shape.

## Procedure

### Dual-Task Paradigm

The dual-task paradigm consisted of a primary task: sentences recognition in noise, and a secondary task: visual shape-matching. Both primary and secondary tasks were performed initially as single tasks, and then simultaneously, as a dual-task.

*Single task*: A. At the beginning of the experiment, the shape-matching visual-motor task (secondary task) was performed for one minute, to familiarize the participants with the task. This time period allowed for presenting 25-36 shape-matching items. Participants were instructed to select and touch the matched shape as quickly and correctly as possible. Correct shape-matching and reaction time for each item were collected by the software. Following the practice trial, the shape-matching task was repeated for three minutes, allowing for presenting 70-105 items in order to equal the duration of each run of Matrix sentences. In keeping both primary and secondary tasks length identical, consistency across all test conditions was accomplished.

B. In the next stage, the Matrix sentences in noise was administered (primary task). Each Matrix sentences run consisted of 20 sentences, mixed with speech-shaped noise, presented at 60dBSPL, in initial SNR of 0dB. Participants were instructed to listen to each sentence and repeat aloud each word, as correctly as possible. Correct recognition of each word in a sentence led to a decrease in sentences intensity-level in relation to the noise intensity-level, thus decreasing SNR., whereas incorrect recognition led to an increase in sentences level, thus increasing the SNR. The first step-size was 3dB, followed by an exponential decrease in step-size, after each reversal of the presentation level. In the end, the speech reception threshold (SRTn) was calculated using the maximum likelihood method (Brand and Kollmeier, 2002). SRT 80% was obtained for each 20 sentence run. Participants performed three lists of 20 sentences due to the known training effect of the Matrix test (Kollmeier et al., 2015; Bugannim et al., 2019). As recommended by Kollmeier et al. (2015), each participant, being a naïve user of the test, performed two training lists of 20 sentences, and the speech reception threshold in noise (SRTn) was determined based on performance of the third list.

*Dual-task*: Subsequent to both single tasks performance, participants performed shape-matching and sentence recognition concurrently, instructed to give priority to the sentence’s recognition task. Matrix sentences in noise were presented to each participant at the SRT 80% that was pre- determined at the single task trial. Stated differently, each participant performed the primary task in the dual-task condition at the SNR that yielded 80% recognition in the single task condition. Thus, listening conditions were fitted individually to participants ability of speech recognition in noise.

### Data Analysis

**FIGURE 1:**
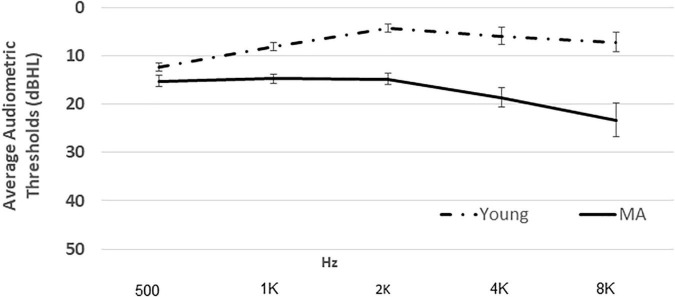
Mean (± sd) air conduction thresholds for the young and middle-aged groups.

All statistical analyzes were carried out using the IBM Statistical Package for the Social Sciences (SPSS) software version 27.0 for Windows. Descriptive statistics for the variables (Mean, SD) were calculated. Age-group characteristics of SRTn required to meet the 80% performance criterion, as well as correct-sentence-recognition in the dual-task, were compared using an independent-samples *t*-test. Next, a mixed model two-way ANOVA was performed with task (single vs. dual) as the within-subject variable and age-group (young vs. MA) as the between-subjects variable. Although hearing thresholds were normal for age for all participants (Figure 1), a comparison of the means of thresholds at 0.5, 1, 2, 4, and 8 kHz, yielded a significant difference, paired samples *t*-test, *t*(38) = −8.43, *p* < 0.001), *d* = 4.72. Consequently, we repeated each analysis including hearing threshold mean as a covariate in addition to the main and interaction effects of the research variables. Finally, following Salthouse and Somberg (1982), in order to control for individual differences in reaction time, as well as initial longer reaction times attributed to age (Meijer et al., 2009) already in the single task, we computed proportional dual-task cost (pDTC) using the following computation: RT pDTC = (RT single task - Rt dual-task)/RT single task X 100.

## Results

**FIGURE 2:**
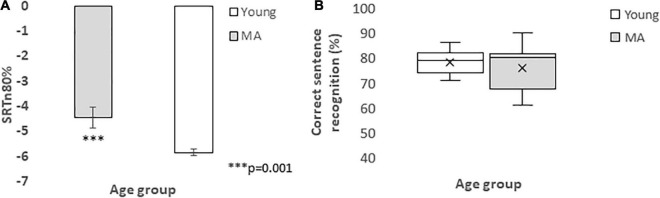
in panel **(A)**, mean (± se) of SRTn 80% sentence recognition of young (white) and MA (gray) groups, in the *single* task. Note that the MA group needed a more positive SNR than the young group, even when the primary task was performed singly. In panel **(B)** distribution of correct sentence recognition for the young (white) and MA (gray), in the *dual-task*, performed at the individual SNR, obtained when tested singly. Lower and upper box boundaries represent the 25th-75th percentiles, lower and upper error bars represent 10th and 90th percentiles, respectively. The horizontal line inside the box represents the median and the X – the mean. Note the larger distribution of the MA results, albeit almost similar median and mean scores.

The auditory single task measure was the SRTn required to meet the 80% performance criterion, As can be seen in Figure 2A, middle-aged adults needed a better SNR (−4.4dB ± 0.43) compared to the young adults (−5.84dB ± 0.13). This result was found significant, in a paired-samples *t*-test, *t*(38) = −3.49, *p* = 0.001, consistent with previous research demonstrating the effect of age on SNR (Desjardins and Doherty, 2013; Degeest et al., 2015; Ward et al., 2017). On the other hand, when the individual SNR was provided in the dual-task to each participant, performance in the young group was, on average, 78.13% (± 0.9) and 76.06% (± 2.1) for the MA, as presented in Figure 2B. The difference between the groups was found insignificant, with *t*(38) = 0.94, *p* = 0.35. This finding suggests the efficiency of the study specific paradigm to manifest LE in the dual-task measures of visual accuracy and reaction time.

**FIGURE 3:**
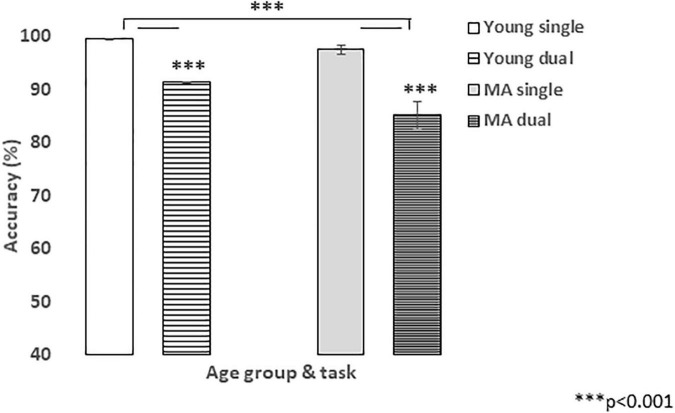
Mean (± se) of visual task accuracy in the single (solid) and dual (pattern) tasks for Young (white) and MA (gray) groups.

Figure 3 presents the means and standard errors for visual accuracy in the single and dual-tasks in the young and middle-aged groups. It can be seen that in both groups the accuracy decreased in the dual-task, from an average of 99.61% (± 0.14) to 91.38% (± 1.16) and from 97.56% (± 0.9) to 85.15% (± 2.4) in the young and middle-aged groups, respectively. ANOVA performed on these data revealed a significant main effect of task with *F* (1,38) = 91.91, *p* < 0.001, η^2^ = 0.71, indicating the presence of LE in the sample. In addition, significant effect was obtained for age-groups, with *F*(1, 38) = 7.57, *p* = 0.009, η^2^ = 0.17. The task X age interaction effect, however, was not significant *F*(1, 38) = 3.80, *p* = 0.059. Furthermore, adding to the analysis the variable of hearing thresholds as a covariate resulted in cancelation of the age-group main effect *F*(1,37) = 0.55, *p* = 0.46, whereas the task main effect persisted, *F*(1,37) = 7.69, *p* = 0.009, η^2^ = 0.17. Thus, although a dual-task effect was obtained for visual accuracy, no age differences emerged for this effect.

**FIGURE 4:**
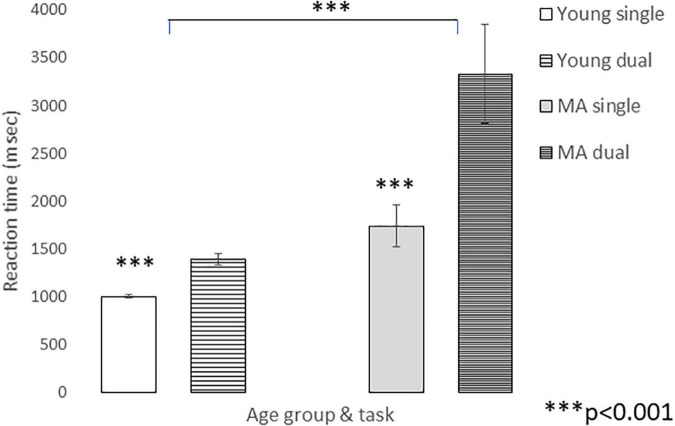
Mean (± se) of visual reaction time in the single (solid) and dual (pattern) tasks for Young (white) and MA (gray) groups.

Figure 4 depicts mean and standard errors for reaction time in the single and dual-tasks, in both groups. Prolongation in reaction time was evident for both groups, however, it was larger for the middle-aged. Whereas in the young group reaction time was prolonged from an average of 1,007.45msec (± 17.99) to an average of 1,391.04msec (± 57.01), in the middle-aged group the average for the single task was 1,742.77msec (± 217.33), while the average for the dual-task was 3,332.3msec (± 510.17). Statistical analysis indicated a significant main effect for task, with *F*(1,38) = 32.82, *p* < 0.001, η^2^ = 0.17, underscoring the difficulty of dual vs single task. Furthermore, the greater prolongation that characterized the middle-aged group, as compared to the young group, was found significant as well, *F*(1, 38) = 20.98, *p* < 0.001, η^2^ = 0.17. In addition, the task X age interaction effect was significant *F*(1, 38) = 12.26, *p* = 0.001, η^2^ = 0.17. After the hearing threshold variable was added as a covariate to the ANOVA model, the main effect of task remained significant, *F*(1, 37) = 4.77, *p* = 0.035)], as well as the main effect of age-group, *F*(1,37) = 10.89, *p* = 0.002, η^2^ = 0.23; and the task X age-group interaction, *F*(1, 37) = 5.62; *p* = 0.023, η^2^ = 0.13. Thus, middle-aged adults exhibited a greater difficulty in the dual-task, irrespective of their hearing status. Notably, the calculation of the RT pDTC in both age-groups yielded a larger pDTC for the MA adults compared to the young adults: 0.95 ± 0.99, and 0.39 ± 0.30, respectively.

## Discussion

The present study aimed at setting a clinical paradigm for the assessment of LE in order to incorporate into the audiological evaluation an important marker of cognitive hearing. Whereas LE manifested by a dual-task paradigm has been a subject of ample research, no specific paradigm was suggested as suitable for the audiology clinic, despite the agreement upon the need of LE measure within the hearing evaluation and intervention framework (Bernarding et al., 2013; Houben et al., 2013; Pals et al., 2015; Alhanbali et al., 2019).

In order to meet the study criterion of 80% correct sentence recognition in the single task, MA adults needed a better SNR of 1.4dB compared to the young adults. In line with previous research (Helfer and Freyman, 2014; Degeest et al., 2015; Dubno, 2015; Helfer, 2015), this finding underscores the known difficulties of MA adults to process speech in noise. Several explanations were offered in the literature to the reduced speech perception ability in the presence of normal hearing acuity. Auditory temporal capabilities were found to influence speech perception in noise capacity in the presence of normal hearing threshold (Helfer and Vargo, 2009; Füllgrabe, 2013), as well as the contribution of elevated extended high frequency thresholds (see review by Helfer and Jesse, 2021). Thus, even though hearing acuity of the participants in the current study was age-appropriate (Engdahl et al., 2005), when compared to the young participants, differences were found significant, and might have inflicted upon the correct sentence recognition of the MA adults. Notwithstanding the peripheral domain influence, Besser et al. (2015) stressed the integration between the auditory and the cognitive systems, suggesting that changes in one domain are associated with changes in the other domain. This notion of the auditory-cognitive association is manifested in our LE results. Despite better SNR, our results indicated that LE was expended by MA adults in the dual-task, more than by young adults, revealed by both measures of the secondary task. Our findings are in line with the few studies that explored LE specifically in MA adults. Degeest et al. (2015) reported increase in LE at the fifth decade of life (SNR dependent). Cramer and Donai (2019) found increased LE in 40-55 years old participants, compared to participants aged 18-25 years. In a related study, a measure of cognitive load was found to increase in normal hearing 51-61 years old participants by Xia et al. (2015). Taken together, our data show that LE increases already in the middle age. Furthermore, consistent with previous studies (Degeest et al., 2015; Cramer and Donai, 2019), our finding of different SNR needed for the 80% correct recognition, suggests that performance accuracy of speech recognition in noise does not fully manifest MA adults’ efforts to maintain successful recognition. The need to allocate more resources than young adults, albeit better listening conditions, and unrelated to hearing thresholds, supports the idea that other auditory processing factors, supra threshold, or otherwise different, affect performance in noise.

Setting a clinical paradigm for the assessment of LE in MA adults might face some hurdles. First, the selection of the appropriate secondary task has been controversial in the literature. Our findings show that using a simple, non-auditory secondary task, resulted in the manifestation of LE in young as well as in MA adults. Both secondary task measures: performance accuracy and reaction time indexed LE. This finding is in line with previous research that used a different modality secondary task, such as tactile (Fraser et al., 2010), or visual (Hughes and Galvin, 2013). In these studies, decreased performance of secondary task measures was evident, regardless of task difficulty. On the other hand, our findings differ from that of Picou and Ricketts (2014), that argued in favor of depth of processing, in order to determine LE by secondary task measures. Trying to solve this contention, it has been suggested that engaging attentional resources across modalities instead of drawing on the same modality, might better reflect LE (Grieco-Calub et al., 2017). Taken together, the use of visual, secondary task in the current study allowed for resources allocation, and expressed LE effectively. Notably, MA adults that visit the audiology clinic are not pre-screened for cognition, thus LE assessment using a simple task might be more beneficial, meeting various cognitive capacities of the MA adults.

Another hurdle in the appropriate paradigm of LE assessment in the MA is the use of RT as a measure of LE. It has been previously established that aging, in general, is related to slower processing of information (Salthouse, 2000), thus RT might not manifest LE in MA adults, being already prolonged in the single task compared to the young adults. Instead, our data demonstrated RT dual-task effect in both groups: young and MA, more so for the MA. Furthermore, in order to overcome individual differences in baseline reaction time, that might be affected by age, we calculated pDTC, and found a RT pDTC, in both groups, more pronounced for the MA. This finding is consistent with Gosselin and Gagné (2011) that showed pDTC in both word and tactile accuracy in older adults. Taken together, it is suggested that MA adults, comparable to older adults, prolonged their responses, more than young adults, to maintain accuracy in the primary task. The cognitive load of speech recognition in noise while matching visual-colored shapes burdened their processing ability, and compelled them to slow their responses. On the whole, this finding proposes the compatibility of the paradigm to assess LE in young and MA adults. Additional studies will need to address other age-groups such as older adults, as well as hearing impaired listeners.

Finally, in an attempt to find a suitable measure for clinical evaluation of LE, physiological measures should be considered as well. One such measure, pupillometry, was found as a measure of cognitive processing load, sensitive to difference in noise types and intelligibility levels (Koelewijn et al., 2012). Moreover, Karatekin et al. (2004) proposed that pupillometry can present the magnitude of resource allocation, and not only the yielding of cognitive capacities. It should be noted, however, that such a measure necessitates appropriate and costly equipment, and might be complicated and inconvenient for the hearing clinics. The current study dual-task paradigm, on the other hand, while reflecting the different proportions of resource allocation by MA compared to young adults, does not require any special equipment other than that found already in the typical hearing clinics. The paradigm is easy to explain, understand, and use, with a time duration of approximately 20 min. Clinicians might find the paradigm helpful, specifically in cases of patients that are not fully aware of the effort they exert in order to understand speech in background noise. These patients sometimes are reluctant to use remote microphone systems or hearing aids. LE assessment might help to encourage them to use such means.

## Limitations

The current study demonstrated the compatibility of a specific dual-task paradigm to manifest LE in MA adults. In order to further substantiate the clinical sensitivity of the paradigm, more participants in the MA, as well as in older adults, are needed. Furthermore, the young adults group consisted of female-only participants. Future studies might consider a mixed-gender group.

In addition, we did not incorporate cognitive tests in the study, as patients coming to the audiological clinic are not pre-screened for cognition. Future studies including cognitive tests may offer the possibility to identify specific aspects of cognitive capacity associated with LE, and further elucidate LE trajectories. Likewise, a self-report LE tool might shed light on strategies listeners use to meet different aspects of listening demands, delineating the cognitive load they are burdened with.

## Conclusion

The current study proposed a clinical tool to assess LE. The dual-task paradigm, using a non-auditory secondary task was found compatible for the assessment of LE in normal hearing young, and more so in MA adults. Hearing thresholds, though significantly different between the two groups, did not account for the greater LE that was manifested in the MA group. Incorporating such a paradigm in the routine clinical setting will address MA adults’ subjective reports, while taking into consideration that successful communication is more than audibility and speech intelligibility.

## Data Availability Statement

The raw data supporting the conclusions of this article will be made available by the authors, without undue reservation.

## Ethics Statement

The studies involving human participants were reviewed and approved by the Institutional Review Board at Tel Aviv University. The patients/participants provided their written informed consent to participate in this study.

## Author Contributions

RKN and CM designed the study. RKN supervised the data collection and organized the database. IR performed the statistical analysis. All authors contributed to manuscript revision, read, and approved the submitted version.

## Conflict of Interest

The authors declare that the research was conducted in the absence of any commercial or financial relationships that could be construed as a potential conflict of interest.

## Publisher’s Note

All claims expressed in this article are solely those of the authors and do not necessarily represent those of their affiliated organizations, or those of the publisher, the editors and the reviewers. Any product that may be evaluated in this article, or claim that may be made by its manufacturer, is not guaranteed or endorsed by the publisher.
